# Assessment of resampling methods for causality testing: A note on the US inflation behavior

**DOI:** 10.1371/journal.pone.0180852

**Published:** 2017-07-14

**Authors:** Angeliki Papana, Catherine Kyrtsou, Dimitris Kugiumtzis, Cees Diks

**Affiliations:** 1 Department of Economics, University of Macedonia, Thessaloniki, Greece; 2 CAC IXXI-ENS Lyon, Lyon, France; University of Paris 10, Paris, France; University of Strasbourg, BETA, Strasbourg, France; 3 Department of Electrical and Computer Engineering, Aristotle University of Thessaloniki, Thessaloniki, Greece; 4 Center for Nonlinear Dynamics in Economics and Finance (CeNDEF), Amsterdam School of Economics, University of Amsterdam, Amsterdam, The Netherlands; Tianjin University, CHINA

## Abstract

Different resampling methods for the null hypothesis of no Granger causality are assessed in the setting of multivariate time series, taking into account that the driving-response coupling is conditioned on the other observed variables. As appropriate test statistic for this setting, the partial transfer entropy (PTE), an information and model-free measure, is used. Two resampling techniques, time-shifted surrogates and the stationary bootstrap, are combined with three independence settings (giving a total of six resampling methods), all approximating the null hypothesis of no Granger causality. In these three settings, the level of dependence is changed, while the conditioning variables remain intact. The empirical null distribution of the PTE, as the surrogate and bootstrapped time series become more independent, is examined along with the size and power of the respective tests. Additionally, we consider a seventh resampling method by contemporaneously resampling the driving and the response time series using the stationary bootstrap. Although this case does not comply with the no causality hypothesis, one can obtain an accurate sampling distribution for the mean of the test statistic since its value is zero under H_0_. Results indicate that as the resampling setting gets more independent, the test becomes more conservative. Finally, we conclude with a real application. More specifically, we investigate the causal links among the growth rates for the US CPI, money supply and crude oil. Based on the PTE and the seven resampling methods, we consistently find that changes in crude oil cause inflation conditioning on money supply in the post-1986 period. However this relationship cannot be explained on the basis of traditional cost-push mechanisms.

## Introduction

Connectivity analysis of multivariate time series is a rapidly growing branch of interest with applications in different fields, such as economy, climatology and brain dynamics. A variety of methods have been developed that uncover complex dynamical structures, i.e. analysis of complex networks from multivariate time series [1–3] and characterization of the complexity of multivariate time series using entropy measures [4, 5].

The investigation of the causal relationships between the variables of a multivariate dynamical system or stochastic process allows us to better understand its structure. In the estimation of direct causality, effects from the remaining variables should be taken into account. We note that by causality we mean Granger causality, either linear and/or nonlinear.

Various causality measures have been recently developed based on information theory. Their advantage is that they are model-free and detect both linear and nonlinear causal effects. The transfer entropy (TE) is the most popular information causality measure, a non-parametric measure that quantifies the amount of information transferred between two random processes [6]. The TE has been proven to be equivalent to the standard linear Granger causality for Gaussian variables [7]. The TE is extended to the partial transfer entropy (PTE) that estimates direct causal effects in multivariate time series [8, 9], and they both have been modified to work on symbol or rank vectors derived from the time series measurements [10–14]. The TE and its variants have been used to investigate the coupling in complex systems [15], also in combination with other methods [16]. In order to avoid the curse of dimensionality, progressive selection of lagged variables [17, 18] and graphical models [19] have been combined with information causality measures. Information causality measures are mainly applied to neuroscience and physiology (e.g. see [20, 21]), as well as finance (e.g. see [22]).

Theoretically, a causality measure should be zero if there are no causal interactions and positive otherwise. However, its value may deviate from the true value (bias) due to the estimation method, the selection of parameters, the finite sample size, the level of noise, as well as the system complexity. In particular for the TE and PTE, the bias can be large stemming from the estimation of conditional densities [23]. In the presence of varying bias, a significance test is more appropriate, than arbitrary thresholding, for deciding the presence of weak coupling. This issue is particular relevant when constructing causality networks with binary connections from direct Granger causality on real multivariate time series [24–28]. Different randomization and bootstrap methods have been employed to correct the bias of the causality measures (see e.g. [29]) and more specifically of transfer entropy (see e.g. [14, 30]).

When using linear Granger causality measures, their asymptotic distribution under the null hypothesis H_0_ of no causal effect is known [31–33]. For information causality measures, parametric tests are only developed when the time series are discrete-valued [13, 34]. When the asymptotic distribution of a test statistic cannot be established, resampling techniques are employed for the construction of its empirical null distribution. The resampled time series should satisfy the H_0_ and also capture the statistical properties of the original time series. Resampled time series can be generated by bootstrapping and randomization.

Bootstrapping is a statistical technique introduced in [35] that aims to estimate the properties of a test statistic when sampling from an approximate distribution. The empirical distribution of the statistic is formed by its values computed on samples drawn with replacement from the original sample. For time series, bootstraps must be carried out in a way that they suitably capture the dependence structure of the data generation process consistent to the H_0_, and be otherwise random (see e.g. [36–38]). For our hypothesis testing, the bootstraps are used to form the null distribution of the causality measure. In a bivariate setting, this is done by bootstrapping the two variables independently or contemporaneously or by only bootstrapping one of the two variables [39].

Statistical tests based on randomization utilize randomized data, which are shuffled samples of the original data, to empirically estimate the expected probability distribution of the estimator. The randomization methods are designed to preserve the dependence structure consistent with H_0_, when randomly shuffling the time series. The time-shifted surrogates [40], as well as other types of surrogates such as the twin surrogates [41], have been extensively suggested in applications, e.g. see [42–45].

In this work, we make an explorative study on resampling time series for the H_0_ of no causal effect and compare seven resampling techniques with regard to the size and power of the significance test, using the PTE as test statistic. Specifically, we combine two resampling techniques: 1) the time-shifted surrogates [40] and 2) the stationary bootstrap [38], with three independence settings of the time series adapted for the non-causality test (giving six resampling methods): A) resampling only the time series of the driving variable, B) resampling independently the driving and the response time series, and C) resampling separately the driving and the response time series, while destroying the dependence of the future and past of the response variable. To the best of our knowledge, schemes B) and C) in conjunction with randomization or bootstrap have not been considered in any methodological study or application. We also introduce a new (seventh) method by bootstrapping contemporaneously the driving and the response time series. In this case, the bootstrap PTE values are centered to zero since the H_0_ of no causal effects is not satisfied.

The empirical distribution of PTE, as well as the size and power of the significance test, for the seven resampling methods are assessed in a simulation study. Some first results on the aforementioned resampling methods have been already presented in [46] and [47]. Here, we extend the study of the examined resampling methods in order to establish their performance.

Finally, to demonstrate the performance of PTE in conjunction with the seven resampling methods using real data, we investigate the possible sources of the US inflation in the post-Volcker era utilizing two 3-variate systems built on the Consumer Price Index for All Urban Consumers, the core CPI, the money supply and the price of crude oil. Empirical results support evidence in favor of a statistically significant direct causal relationship between oil prices and US inflation obeying dynamics which are not comparable with the oil episodes occurred in the 1970s.

## Materials and methods

### Partial transfer entropy

The TE quantifies the amount of information explained in a response variable *Y* at one time step ahead from the state of a driving variable *X* accounting for the concurrent state of *Y*. Let {*x*_*t*_, *y*_*t*_}, *t* = 1, …, *n* be the observed time series of two variables. We define the reconstructed state space vectors of the variables as **x**_*t*_ = (*x*_*t*_, *x*_*t* − *τ*_, …, *x*_*t* − (*m* − 1)*τ*_)′ and **y**_*t*_ = (*y*_*t*_, *y*_*t* − *τ*_, …, *y*_*t* − (*m* − 1)*τ*_)′, where *m* is the embedding dimension and *τ* the time delay. The TE from *X* to *Y* constitutes the conditional mutual information *I*(*y*_*t*+1_; **x**_*t*_|**y**_*t*_) given as [6]

$$\begin{array}{ccc} {\text{TE}}_{X\to Y} & = & I\left(y_{t+1};x_{t}|y_{t}\right)=\sum p\left(y_{t+1},x_{t},y_{t}\right)\log\frac{p(y_{t+1}|x_{t},y_{t})}{p(y_{t+1}|y_{t})}\\ & = & H\left(x_{t},y_{t}\right)-H\left(y_{t+1},x_{t},y_{t}\right)+H\left(y_{t+1},y_{t}\right)-H\left(y_{t}\right), \end{array}$$ (1)

where TE is expressed either based on the probability distributions, *p*(⋅) (here being defined for the discretized variables), or the entropy terms, *H*(⋅), where *H*(**x**) = −∫*f*(**x**)log *f*(**x**)*d***x** is the differential entropy of the vector variable **x** with probability density function *f*(**x**). We note that *m* and *τ* are set to be similar for both variables as suggested in [29].

The partial transfer entropy (PTE) is the multivariate extension of transfer entropy (TE) in [8, 9]. The PTE accounts for the direct coupling of *X* to *Y* conditioning on the remaining variables of a multivariate system, collectively denoted by *Z*. It is defined as

$$\begin{array}{ccc} {\text{PTE}}_{X\to Y|Z} & = & I(y_{t+1};x_{t}|y_{t},z_{t})\\ & = & H\left(x_{t},y_{t},z_{t}\right)-H\left(y_{t+1},x_{t},y_{t},z_{t}\right)+H\left(y_{t+1},y_{t},z_{t}\right)-H\left(y_{t},z_{t}\right). \end{array}$$ (2)

The estimation of PTE relies on the estimation of the joint probability density functions in the expression of the entropies. Different types of estimators for the TE and PTE exist, such as histogram-based (e.g. by discretizing the variables to equidistant intervals [48]), kernel-based [49] and using correlation sums [50]. In this paper, we choose the nearest neighbor estimator [51], which is specifically effective for high-dimensional data [18]. This estimator uses the distances between the reconstructed state space vectors to estimate the joint and marginal densities. For each reference point, viewed in the largest state space, the distance length *ϵ* is defined as the distance to the *k*-th nearest neighbor. Then densities, at projected subspaces, are locally formed by the number of points within *ϵ* from each reference point. Thus, the free parameter in the estimation of entropies is the number of neighbors *k*.

Theoretically, the causality measures including the PTE should be zero in the case of no causal effects. However, various issues such as the estimation method for the entropies and subsequently densities, the selection of the embedding parameters, the finite sample size and the inherent dynamics of each subsystem [29] may introduce bias. In order to determine whether a PTE value indicates a weak coupling or it is not statistically significant, resampling methods are employed.

### Resampling methods

Our examined null hypothesis H_0_ is that there is no direct causal effect from *X* to *Y* or more specifically that PTE_*X* → *Y*|*Z*_ = 0, i.e. *I*(*y*_*t*+1_; **x**_*t*_|**y**_*t*_, **z**_*t*_) = 0. In order to generate resampled time series representing the H_0_, we consider two resampling techniques, i.e. 1) the time-shifted surrogates and 2) the stationary bootstrap, and combine them with three independence settings. Thus six resampling methods (cases 1A to 2C) are formulated to test the H_0_. In addition, we introduce a seventh resampling method that is based on the stationary bootstrap and does not directly comply with the H_0_.

#### Resampling techniques

**1**) **Time-shifted surrogates**. Let us consider two variables *X* and *Y* and their corresponding time series {*x*_1_, …, *x*_*n*_} and {*y*_1_, …, *y*_*n*_}. The time-shifted surrogates are generated, so that they preserve the dynamics of the original time series, i.e. {*x*_1_, …, *x*_*n*_}, while the couplings between *X* and *Y* are destroyed [40]. They are formed by cyclically time-shifting the components of a time series. In more details, for the time series {*x*_1_, …, *x*_*n*_}, an integer *d* is randomly chosen and the *d* first values of the time series are moved to the end, giving the time-shifted surrogate time series $\left\{x_{t}^{*}\right\}=\left\{x_{d+1},\ldots ,x_{n},x_{1},\ldots ,x_{d}\right\}$. The random number, *d*, is randomly drawn from the discrete uniform distribution in the range [0.05*n*; 0.95*n*] in order to maintain disruption of the time order of the original time series even in the presence of strong autocorrelation.

**2**) **The stationary bootstrap**. The stationary bootstrap was introduced in [38] to adapt bootstrap on correlated data. By construction, the stationary bootstrap does not destroy the time dependence of the data. This method tries to replicate the correlations by resampling blocks of data. The lengths of the resampled blocks have a geometric distribution. For a fixed probability *p*, block lengths *L*_*i*_ are generated with probability *p*(*L*_*i*_ = *k*) = (1 − *p*)^(*k* − 1)^ *p* for *k* = 1, 2, …. The starting time points of the blocks *I*_*i*_ are drawn from the discrete uniform distribution on {1, …, *n* − *k*}. A bootstrap time series $\left\{x_{t}^{*}\right\}$ is formed by first starting with a random block as defined above *B*_*I*_1_, *L*_1__ = {*x*_*I*_1__, *x*_*I*_1_+1_, …, *x*_*I*_1_+*L*_1_ − 1_}, and blocks are added until length *n* is reached.

#### Independence settings

The three independence settings presented below regard both time-shifted surrogate and stationary bootstrapped time series.

**A**. The first setting is to resample only the time series of the driving variable *X*. This constitutes the standard approach for the surrogate test for the significance of causality measures [18, 40, 52, 53]. The intrinsic dynamics of the variable *X* is preserved in the resampled time series $\left\{x_{t}^{*}\right\}$ but the coupling between X* and *Y* is destroyed. So, H_0_ is approximated and PTE_X* → *Y*|*Z*_ = 0. The variables *X* and *Y* as well as *X* and *Z* are independent, however the pair of variables (*Y*, *Z*) preserves its interdependence.

**B**. This second scheme resamples both the driving variable *X* and the response variable *Y*, i.e. the resampled time series $\left\{x_{t}^{*}\right\}$ and $\left\{y_{t}^{*}\right\}$ are generated. Again, the intrinsic dynamics of both *X* and *Y* are preserved but the coupling between them is destroyed, so that PTE_X* → Y*|*Z*_ = 0. Here, independence holds for all variable pairs (*X*, *Y*), (*Y*, *Z*) and (*X*, *Z*). Nevertheless, there is still no complete independence between all arguments in the definition of PTE, as *y*_*t*+1_ preserves by construction of $\left\{y_{t}^{*}\right\}$ its dependence on **y**_*t*_.

**C**. The third scheme establishes complete independence of all the terms involved in the definition of PTE, i.e. in addition to the resampling of *X* and *Y*, also *y*_*t*+1_ is resampled separately. Technically, we first form the reconstructed vectors of *X* and *Y* and then we randomly shuffle them independently for each time series. In this way, the time dependence is destroyed between *y*_*t*+1_, **x**_*t*_ and **y**_*t*_ and therefore they become independent. Further, **z**_*t*_ becomes independent of **x**_*t*_, **y**_*t*_ but not of *y*_*t*+1_.

The seventh resampling method uses stationary bootstrap to resample contemporaneously the driving and the response time series (*X*, *Y*). The resampled time series are not consistent to H_0_ because the coupling of *X* and *Y* is not destroyed. In order to obtain an accurate sampling distribution of the mean of the test statistic one can take into consideration that the mean value of the test statistic is zero under H_0_. The idea is that $\sqrt{n}$(PTE—*E*(PTE)), where *E*(PTE) is the mean of PTE, can be distributed similarly for series that comply to H_0_ (*E*(PTE) = 0) and series that do not (*E*(PTE) >0); it is assumed that $\sqrt{n}$(PTE—*E*(PTE)) tends to the normal distribution with zero mean and known variance [38]. Since our goal is to compare the different resampling methods, no results for this approximation of the true distribution are discussed. By centering the distribution of the bootstrap PTE values around zero, we get an approximation of the null distribution of PTE. Thus, this resampling method can be employed to test H_0_, provided that the null distribution of the bootstrap values of the test statistic is shifted to have mean zero. It is labelled as 2D to stress that it the fourth setting for the stationary bootstrap.

## Simulation study

We apply the significance test for the PTE with the seven resampling methods to multiple realizations of various simulation systems. Specifically, we estimate the PTE from 1000 realizations per simulation system. For each realization and each resampling method, *M* = 100 resampled time series are generated. Let us denote *q*_0_ the PTE value from one realization of a system and *q*_1_, *q*_2_, …, *q*_*M*_ the PTE values from the resampled time series for this particular realization and for a specific resampling method. The rejection of H_0_ of no causal effects is decided by the rank ordering of the PTE values computed on the original time series, *q*_0_, and the resampled time series, *q*_1_, …, *q*_*M*_. For the one-sided test, if *r*_0_ is the rank of *q*_0_ when ranking the list *q*_0_, *q*_1_, …, *q*_*M*_ in ascending order, the *p*-value of the test is 1 − [(*r*_0_ − 0.326)/(*M* + 1 + 0.348)], by applying the correction in [54].

The simulation systems we considered in this study are:

1. Three coupled Hénon maps, with nonlinear couplings (*X*_1_ → *X*_2_ → *X*_3_)
  $$\begin{array}{ccc} x_{1,t} & = & 1.4-x_{1,t-1}^{2}+0.3x_{1,t-2}\\ x_{2,t} & = & 1.4-cx_{1,t-1}x_{2,t-1}-\left(1-c\right)x_{2,t-1}^{2}+0.3x_{2,t-2}\\ x_{3,t} & = & 1.4-cx_{2,t-1}x_{3,t-1}-\left(1-c\right)x_{3,t-1}^{2}+0.3x_{3,t-2}, \end{array}$$
  with equal coupling strengths *c* for *X*_1_ → *X*_2_ and *X*_2_ → *X*_3_. We set *c* = 0 (uncoupled case), *c* = 0.3 (moderate coupling) and *c* = 0.5 (strong coupling). We note that the time series of this system become completely synchronized for coupling strengths *c* ≥ 0.7.
2. A vector autoregressive process of 4 variables and order 5, VAR(5), with linear couplings (*X*_4_ → *X*_2_ → *X*_1_ → *X*_3_ and *X*_2_ → *X*_3_)
  $$\begin{array}{ccc} x_{1,t} & = & 0.8x_{1,t-1}+0.65x_{2,t-4}+\epsilon_{1,t}\\ x_{2,t} & = & 0.6x_{2,t-1}+0.6x_{4,t-5}+\epsilon_{2,t}\\ x_{3,t} & = & 0.5x_{3,t-3}-0.6x_{1,t-1}+0.4x_{2,t-4}+\epsilon_{3,t}\\ x_{4,t} & = & 1.2x_{4,t-1}-0.7x_{4,t-2}+\epsilon_{4,t}, \end{array}$$
  where *ϵ*_*i*, *t*_, *i* = 1, …, 4, are independent to each other Gaussian white noise processes with unit standard deviation (Eq (12) in [55]).
3. Five coupled Hénon maps, with nonlinear couplings (*X*_1_ → *X*_2_ → *X*_3_ → *X*_4_ → *X*_5_) defined similarly to system 1. We consider again equal coupling strengths *c*, and set *c* = 0 (uncoupled case), *c* = 0.2 (moderate coupling) and *c* = 0.4 (strong coupling).

We consider two time series lengths: *n* = 512 and 2048. The calculation of the PTE relies on the phase space reconstruction [56, 57]; specifically for PTE see [8]. Since all the simulation systems are discrete in time we set the time delay *τ* equal to one, while the embedding dimension *m* is identical for all variables, which is reported to be the best strategy [29], and for each system it is set according to its complexity, i.e. taking into account the maximum delay in the equations of each system. The number of nearest neighbors for the estimation of the probability distributions equals 10 (the choice of *k* does not substantially affect the estimation of PTE [53, 58]).

To investigate the performance of the significance tests for the PTE with the different resampling methods, we use the sensitivity of the PTE, i.e the percentage of rejection of H_0_ when there is true direct causality, as well as the specificity of the PTE, i.e. the percentage of no rejection of H_0_ when there is no direct causality, at the significance level *α* = 0.05. The notation *X*_2_ → *X*_1_|*Z* denotes the Granger causality from *X*_2_ to *X*_1_, accounting for the presence of confounding variables *Z* = *X*_3_, …, *X*_*K*_, where *K* is the number of observed variables. For brevity, we use the notation *X*_2_ → *X*_1_ instead of *X*_2_ → *X*_1_|*Z*, implying the conditioning on the confounding variables. The same holds for the remaining pairs of variables.

**System 1**. The PTE is negatively biased; the mean PTE values from the 1000 realizations at all directions are negative when *c* = 0 (Table 1). For *c* = 0.3 and *c* = 0.5, it is larger when direct couplings exist (*X*_1_ → *X*_2_, *X*_2_ → *X*_3_) and raises with *n*. Regarding the indirect coupling *X*_1_ → *X*_3_, the PTE slightly increases with *n* as *c* increases, reaching the highest mean value for *c* = 0.5 (mean PTE_*X*_1_ → *X*_3__ = 0.0004 for *n* = 512 and PTE_*X*_1_ → *X*_3__ = 0.0071 for *n* = 2048). For the rest of the couplings, the PTE is negative at the same level regardless of *c* or *n*. The occurrence of many negative values of the PTE indicates the need for a significance test.

**Table 1:** Mean PTE values from 1000 realizations of system 1 for *n* = 512 and 2048, highlighted at the directions of the true couplings.

*n* = 512	*X*_1_ → *X*_2_	*X*_2_ → *X*_1_	*X*_2_ → *X*_3_	*X*_3_ → *X*_2_	*X*_1_ → *X*_3_	*X*_3_ → *X*_1_
*c* = 0	-0.0059	-0.0062	-0.0062	-0.0061	-0.0058	-0.0056
*c* = 0.3	**0.0802**	-0.0042	**0.0885**	-0.0064	-0.0045	-0.0074
*c* = 0.5	**0.2324**	-0.0071	**0.1557**	-0.0044	0.0004	-0.0079
*n* = 2048	*X*_1_ → *X*_2_	*X*_2_ → *X*_1_	*X*_2_ → *X*_3_	*X*_3_ → *X*_2_	*X*_1_ → *X*_3_	*X*_3_ → *X*_1_
*c* = 0	-0.0086	-0.0088	-0.0087	-0.0085	-0.0087	-0.0088
*c* = 0.3	**0.1736**	-0.0024	**0.1725**	-0.0059	-0.0039	-0.0094
*c* = 0.5	**0.3649**	-0.0026	**0.2601**	-0.0049	0.0071	-0.0078

We evaluate how the null distribution of the PTE from the seven resampling methods differs with respect to the original PTE values. For *c* = 0, all of them correctly indicate the absence of couplings as the percentage of rejection at *a* = 0.05 is not larger than 5% (Table 2). Considering *c* = 0.3, the true couplings are identified again. However, spurious and indirect couplings are indicated as well for the setting A and less for B. Additionally, similar performance is observed when the coupling strength is strong (*c* = 0.5) and large percentages are obtained for the indirect coupling *X*_1_ → *X*_3_ in all schemes.

**Table 2:** Percentage of significant PTE values for system 1 for *n* = 512 / 2048, for all resampling methods. A single number is displayed when the same percentage corresponds to both *n*. The true couplings are highlighted.

*c* = 0	*X*_1_ → *X*_2_	*X*_2_ → *X*_1_	*X*_2_ → *X*_3_	*X*_3_ → *X*_2_	*X*_1_ → *X*_3_	*X*_3_ → *X*_1_
1A	5.7 / 4.2	5.6 / 5.2	4.7 / 4.9	5.3 / 5.6	5.8 / 5.5	5.5 / 5.2
1B	5.2 / 4.8	4.6 / 5.6	4 / 5.2	4.3 / 6.6	4.6 / 5	5.8 / 5.5
1C	0.7 / 0	0.8 / 0	0.4 / 0	0.7 / 0	0.3 / 0	0.5 / 0
2A	4.4 / 3.8	3.1 / 3.9	3.4 / 4.1	4.5 / 4.5	4.5 / 4.3	4.1 / 5.1
2B	1.9 / 0.4	1.9 / 0.7	1.8 / 0.6	2.1 / 0.3	1.9 / 0.5	2.4 / 1
2C	0.6 / 0	0.6 / 0	0.3 / 0	0.5 / 0	0.4 / 0	0.1 / 0
2D	0.6 / 0	0.7 / 0	0.3 / 0	0.7 / 0	0.2 / 0	0.2 / 0
*c* = 0.3	**X**_**1**_ → **X**_**2**_	*X*_2_ → *X*_1_	**X**_**2**_ → **X**_**3**_	*X*_3_ → *X*_2_	*X*_1_ → *X*_3_	*X*_3_ → *X*_1_
1A	**100**	11.8/ 40.1	**100**	9.5 / 17.2	12.8/ 34	6.1 / 5.5
1B	**100**	9 / 37.2	**100**	2.7 / 1.8	5.4 / 6.7	5 / 4.3
1C	**100**	0.9 / 0.5	**86.3** / **100**	0	0.2 / 0	0.4 / 0.1
2A	**100**	8.7 / 32.8	**100**	6.9 / 13.5	8.9 / 28	4.7 / 4.1
2B	**100**	2.9 / 13.7	**100**	0.9 / 0.3	1.2 / 1.7	1.2 / 0.5
2C	**100**	0.8 / 0.6	**99.9** / **100**	0	0 / 0.1	0.3 / 0.1
2D	**100**	1.2 / 0.9	**100**	0.1 / 0	0.2 / 0.3	0.4 / 0.1
*c* = 0.5	**X**_**1**_ → **X**_**2**_	*X*_2_ → *X*_1_	**X**_**2**_ → **X**_**3**_	*X*_3_ → *X*_2_	*X*_1_ → *X*_3_	*X*_3_ → *X*_1_
1A	**100**	8.1 / 33.8	**100**	10.2 / 21.5	31 / 96.3	6.2 / 8.3
1B	**100**	4.3 / 30.4	**100**	1.7 / 1.4	9.1 / 67.3	4.5 / 4.8
1C	**100**	0.7 / 0.4	**100**	0	1.9 / 25.4	0.1
2A	**100**	5.1 / 29.2	**100**	7.7 / 17	24.1 / 94.7	4 / 7.1
2B	**100**	2 / 11	**100**	0.8 / 0.2	5.2 / 53.3	1.3 / 0.8
2C	**100**	0 / 0.2	**100**	0	1.2 / 24.3	0 / 0.1
2D	**100**	0.2 / 0.6	**100**	0 / 0.1	1.4 / 11.6	0.1

The sensitivity of PTE is assessed from the two true causal links, i.e. *X*_1_ → *X*_2_ and *X*_2_ → *X*_3_ since we calculate the proportion of ‘positives’ (true causal links) that are correctly identified. A high sensitivity is established by a high percentage of significant PTE values over the 1000 realizations for these two couplings, which means that the PTE correctly detects the true causal effects. Similarly, the specificity of PTE is decided by the percentage of the significant PTE for the remaining couples, for which there is no true causal effect. A low percentage of significant PTE values signifies a large proportion of ‘negatives’ (no causal links) correctly identified.

**Fig 1:**
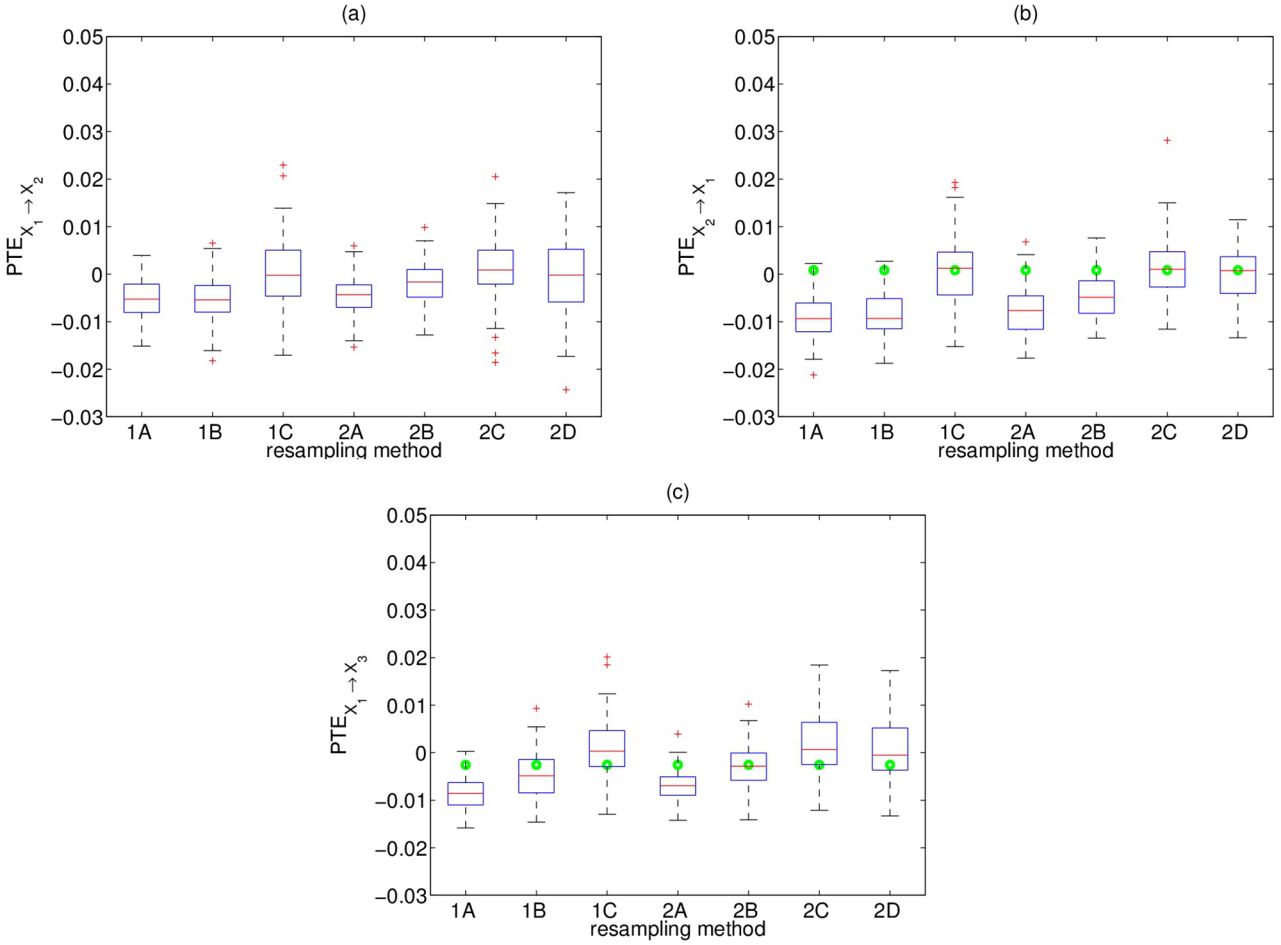
Boxplots of surrogate/bootstrap PTE values and original PTE value from one realization of system 1 for *c* = 0.3 and *n* = 2048, for the directions (a) *X*_1_ → *X*_2_ (direct coupling), (b) *X*_2_ → *X*_1_ (no coupling) and (c) *X*_1_ → *X*_3_ (indirect coupling). The dots at the same level denote the PTE value on the original data, and in (a) the value is 0.19 and not displayed. The central mark, the bottom and top edges on each box indicate the median, the 25th and 75th percentile, respectively. Outliers are denoted with the ‘+’.

Concerning the first six resampling methods, the percentage of erroneously rejected H_0_ for non-existing or indirect couplings tends to increase with *c* and the time series length *n*, the most robust being 1C and 2C. It turns out that when the resampled time series become more independent (from A to C), the percentage of spurious couplings decreases. This is so because the null distribution for the test is somewhat more spread and displaced to the right as the resampling changes from the least independent scheme (setting A) to the most independent one (setting C) (Fig 1).

The resampling method 2D seems to be the most effective one as it attains the same highest percentage of rejection for true direct couplings and the lowest percentage of rejection for no direct coupling. We note that the green dots are not displayed in Fig 1a because they exceed the axis and we kept the same range of PTE values (*y*-axis) in all subfigures in order to be able to straightforwardly compare the different cases.

We are interested in the spread of the resulting surrogate null distribution. Thus, we display some indicative results for the mean value of the means and standard deviations of the surrogate PTE values over all the realizations for the direction *X*_1_ → *X*_2_ and for time series length *n* = 512 in Table 3. The more independent setting we consider (from A to B to C), the greater the median and the mean (as shown in Fig 1 and Table 3, respectively) and the larger the spread of the distribution of the surrogate PTE values, while case 2D features one of the greatest spreads.

**Table 3:** Mean value of all means and standard deviations (std) over all realizations of system 1 of the surrogate PTE values for the direction *X*_1_ → *X*_2_ and time series length *n* = 512 for each resampling case.

*c* = 0	mean	std	*c* = 0.3	mean	std	*c* = 0.5	mean	std
1A	-0.0059	0.0085	1A	-0.0056	0.0075	1A	-0.0037	0.0087
1B	-0.0059	0.0086	1B	-0.0042	0.0100	1B	-0.0009	0.0190
1C	0.0009	0.0107	1C	0.0000	0.0115	1C	0.0008	0.0115
2A	-0.0049	0.0086	2A	-0.0043	0.0076	2A	-0.0025	0.0086
2B	-0.0019	0.0087	2B	-0.0013	0.0079	2B	0.0003	0.0087
2C	0.0022	0.0104	2C	0.0023	0.0113	2C	0.0023	0.0112
2D	0.0000	0.0103	2D	0.0000	0.0135	2D	0.0000	0.0182

**System 2**. The mean PTE values from 1000 realizations of the second system are all positive and the PTE for the directions of the true couplings is larger, with the exception of *X*_2_ → *X*_3_ being at the level of no direct coupling and not significantly increasing with *n* (Table 4). The level of the PTE for the uncoupled directions varies from 0.0014 to 0.0097 and decreases with *n*.

**Table 4:** As Table 1 but for system 2.

	*X*_1_ → *X*_2_	**X**_**2**_ → **X**_**1**_	**X**_**1**_ → **X**_**3**_	*X*_3_ → *X*_1_	*X*_1_ → *X*_4_	*X*_4_ → *X*_1_
*n* = 512	0.0044	**0.0914**	**0.0757**	0.0032	0.0057	0.0038
*n* = 2048	0.0026	**0.1232**	**0.0960**	0.0014	0.0038	0.0021
	**X**_**2**_ → **X**_**3**_	*X*_3_ → *X*_2_	*X*_2_ → *X*_4_	**X**_**4**_ → **X**_**2**_	*X*_3_ → *X*_4_	*X*_4_ → *X*_3_
*n* = 512	**0.0056**	0.0052	0.0097	**0.1002**	0.0069	0.0033
*n* = 2048	**0.0058**	0.0029	0.0064	**0.1348**	0.0045	0.0014

**Fig 2:**
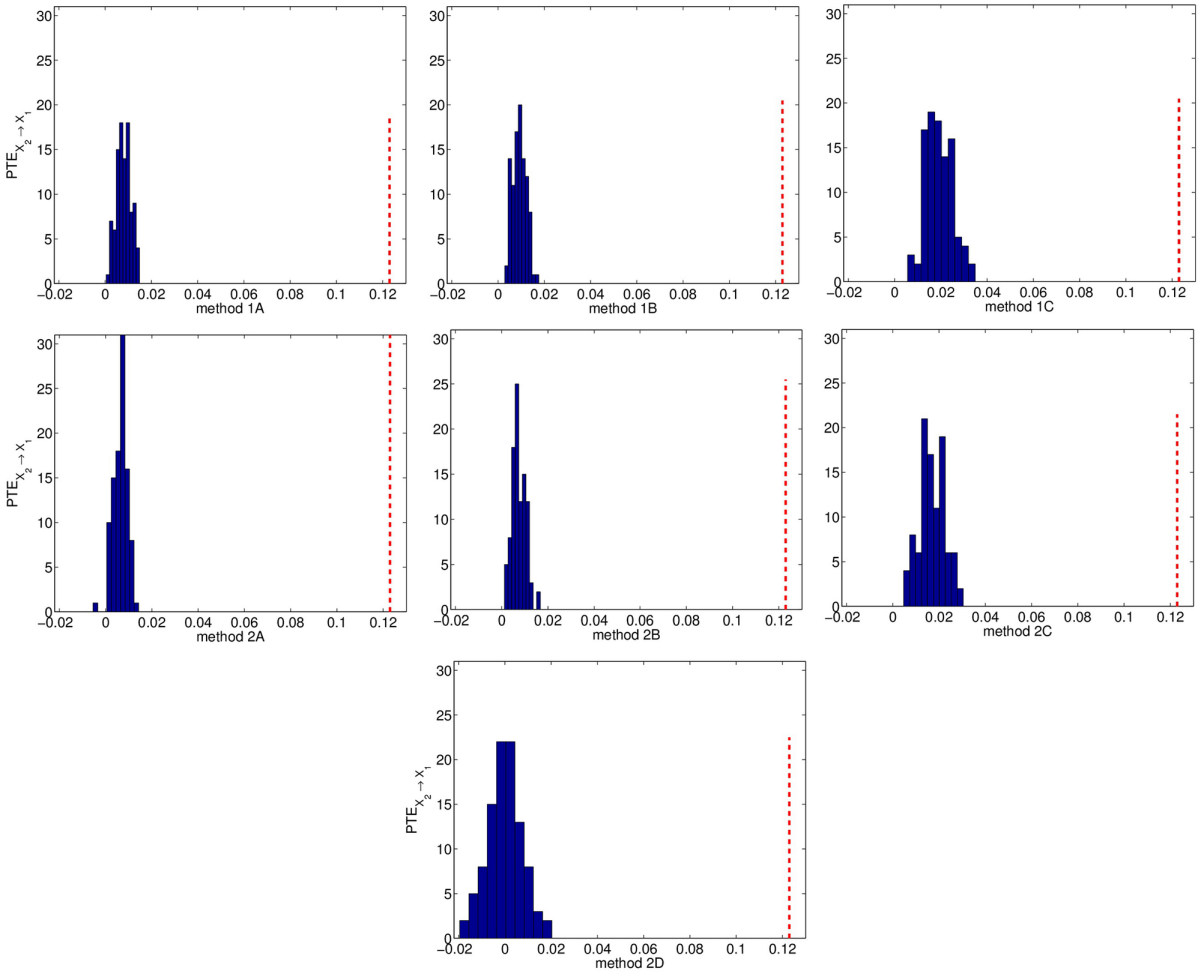
Distribution of surrogate/bootstrap PTE values and original PTE value (vertical dotted line) from one realization of system 2 with *n* = 2048, for the direction *X*_2_ → *X*_1_.

**Fig 3:**
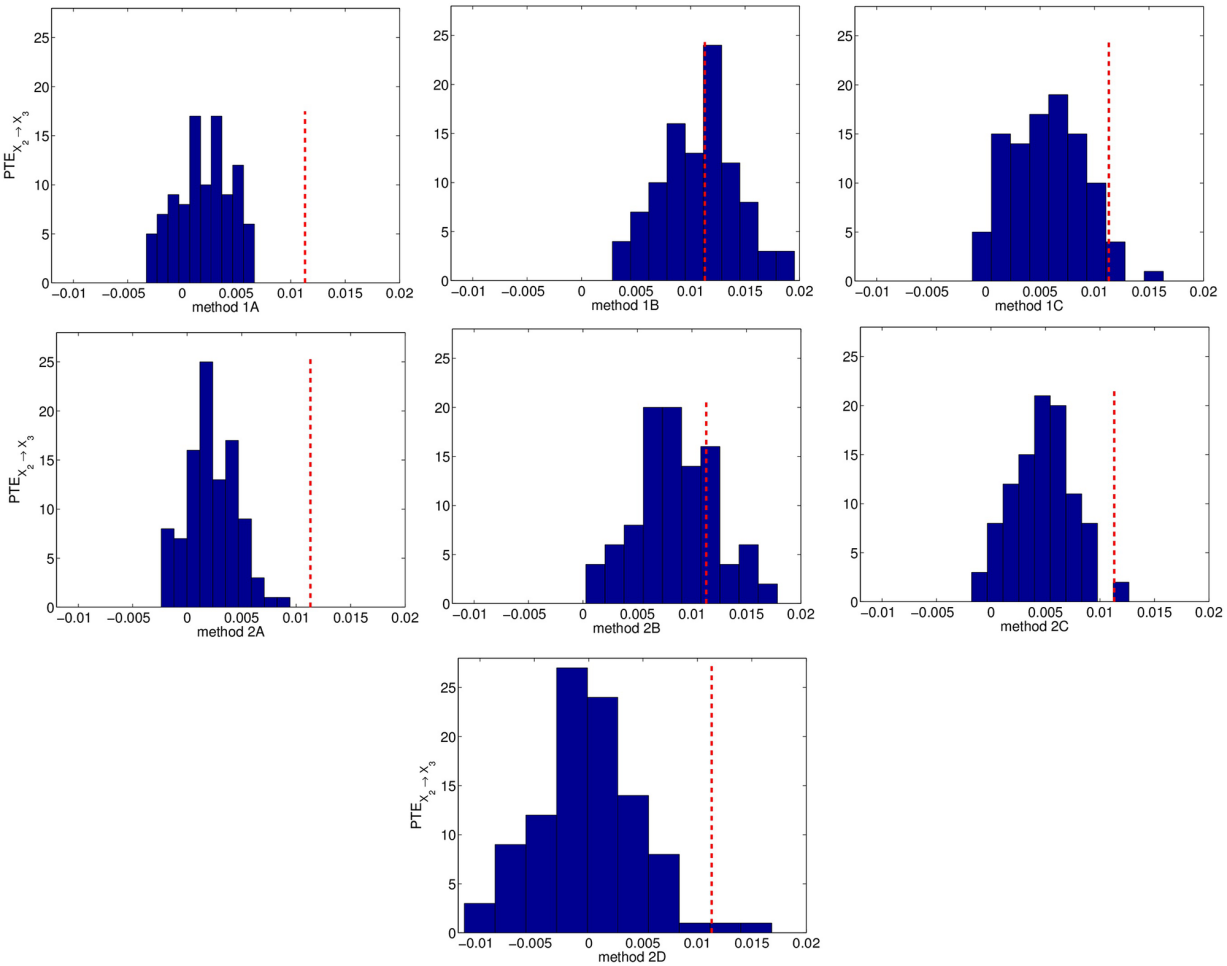
As Fig 2 but for the direction *X*_2_ → *X*_3_.

The true couplings *X*_2_ → *X*_1_, *X*_1_ → *X*_3_, *X*_4_ → *X*_2_ are well established by the significance test (Table 5). The weak coupling *X*_2_ → *X*_3_ is detected only by the setting A (1A and 2A), with the power of the test increasing with *n*. No spurious causalities are identified by the first six resampling methods (percentage of significant PTE varies from 0% to 6% at the uncoupled directions), however method 2D identifies wrongly the couplings *X*_2_ → *X*_4_ and *X*_3_ → *X*_4_, giving much higher percentage than the nominal size 5%. The surrogate/bootstrap PTE values seem to increase as the resampled time series become more independent. This can be clearly observed when comparing settings A and B, as shown in Fig 2 for the strong coupling *X*_2_ → *X*_1_ and Fig 3 for the weak coupling *X*_2_ → *X*_3_. The bootstrap PTE values for method 2D are centered around zero by construction, while the surrogate/bootstrap PTE values for the other six resampling methods are positively biased. Their distribution becomes wider as the resampling method gets more independent (A to C), with method 2D having the wider one. The latter performs poorly because the distribution of the bootstrap PTE values is much wider compared to the other ones and the original PTE value falls within the tail of this distribution (Fig 3, case 2D).

**Table 5:** As Table 2 but for system 2.

	*X*_1_ → *X*_2_	**X**_**2**_ → **X**_**1**_	**X**_**1**_ → **X**_**3**_	*X*_3_ → *X*_1_	*X*_1_ → *X*_4_	*X*_4_ → *X*_1_
1A	0.4 / 0	**100**	**100**	0.6 / 0.3	0.1 / 0	4.6 / 3.2
1B	0	**100**	**99.4** / **100**	0	0	0
1C	0	**100**	**100**	0	0	0
2A	0.4 / 0	**100**	**100**	0.5	0.1 / 0	2.8 / 3.7
2B	0	**100**	**100**	0	0.2 / 0	0 / 0
2C	0	**100**	**99.7** / **100**	0	0	0
2D	2.3 / 3.8	**100**	**100**	0.8 / 0.5	8.2 / 15.7	1.9 / 1.8
	**X**_**2**_ → **X**_**3**_	*X*_3_ → *X*_2_	*X*_2_ → *X*_4_	**X**_**4**_ → **X**_**2**_	*X*_3_ → *X*_4_	*X*_4_ → *X*_3_
1A	**18.8** / **62.4**	1.1 / 0.2	3.5 / 2	**100**	0.8 / 0	6 / 4.2
1B	**0** / **0.1**	0	2.1 / 1.3	**99.9** / **100**	0.4 / 0	0
1C	**3.7** / **10.1**	0	0	**100**	0	0.9 / 0
2A	**11.7** / **60.1**	0.6 / 0.1	2.6 / 3.2	**100**	0.4 / 0	3.1
2B	**0** / **0.2**	0	3.1	**100**	0.8 / 0.1	0
2C	**3.4** / **18.5**	0	0	**100**	0	0.2 / 0
2D	**2.7** / **24**	4.7 / 6.7	21.6 / 37.8	**100**	15.7 / 24.2	0.8 / 0.6

**System 3**. The mean PTE values from 1000 realizations of the third system are presented in Table 6. Slightly negative PTE values are obtained at the uncoupled directions, while some positive ones come up for the directions of the true couplings. Positive values are estimated for large coupling strength and indirect causal effects (e.g. *X*_2_ → *X*_4_), but they are much smaller compared to those for direct causal effects.

**Table 6:** As Table 1 but for system 3.

	*n* = 512			*n* = 2048		
	*c* = 0	*c* = 0.2	*c* = 0.4	*c* = 0	*c* = 0.2	*c* = 0.4
*X*_1_ → *X*_2_	-0.0012	**0.0104**	**0.0510**	-0.0027	**0.0274**	**0.1096**
*X*_2_ → *X*_1_	-0.0014	-0.0009	-0.0042	-0.0028	-0.0009	-0.0003
*X*_1_ → *X*_3_	-0.0010	-0.0019	-0.0011	-0.0028	-0.0033	0.0014
*X*_3_ → *X*_1_	-0.0016	-0.0016	-0.0031	-0.0028	-0.0035	-0.0034
*X*_1_ → *X*_4_	-0.0013	-0.0023	-0.0023	-0.0027	-0.0037	-0.0043
*X*_4_ → *X*_1_	-0.0012	-0.0015	-0.0023	-0.0027	-0.0036	-0.0043
*X*_1_ → *X*_5_	-0.0009	-0.0022	-0.0030	-0.0030	-0.0039	-0.0046
*X*_5_ → *X*_1_	-0.0015	-0.0012	-0.0025	-0.0027	-0.0036	-0.0039
*X*_2_ → *X*_3_	-0.0012	**0.0123**	**0.0576**	-0.0027	**0.0286**	**0.1079**
*X*_3_ → *X*_2_	-0.0012	-0.0002	0.0003	-0.0028	-0.0025	-0.0014
*X*_2_ → *X*_4_	-0.0011	-0.0001	0.0028	-0.0027	-0.0027	0.0028
*X*_4_ → *X*_2_	-0.0015	-0.0011	-0.0020	-0.0029	-0.0034	-0.0036
*X*_2_ → *X*_5_	-0.0008	-0.0009	-0.0003	-0.0029	-0.0035	-0.0026
*X*_5_ → *X*_2_	-0.0012	-0.0010	-0.0017	-0.0029	-0.0035	-0.0039
*X*_3_ → *X*_4_	-0.0009	**0.0135**	**0.0510**	-0.0026	**0.0300**	**0.1015**
*X*_4_ → *X*_3_	-0.0011	0.0004	0.0015	-0.0025	-0.0020	-0.0006
*X*_3_ → *X*_5_	-0.0012	-0.0000	0.0028	-0.0029	-0.0028	0.0034
*X*_5_ → *X*_3_	-0.0010	-0.0005	-0.0002	-0.0027	-0.0032	-0.0026
*X*_4_ → *X*_5_	-0.0009	**0.0122**	**0.0446**	-0.0029	**0.0284**	**0.0928**
*X*_5_ → *X*_4_	-0.0014	0.0002	0.0025	-0.0028	-0.0020	-0.0007

No couplings are found in the uncoupled case (*c* = 0) for system 3 (Table 7). A Table including the percentage of significant PTE values for system 3 for all the directions is available as a Supporting file (S1 Table). The percentage of significant PTE values range from 0% to 5.6% for all the resampling methods and both time series lengths. The PTE is also effective when couplings are present. When *c* = 0.2, its sensitivity increases with *n*, and when *c* = 0.4 the highest sensitivity tends to be obtained even for small *n*.

**Table 7:** As Table 2 but for system 3 for the true couplings, an indirect coupling (*X*_2_ → *X*_4_) and an uncoupled case (*X*_5_ → *X*_4_).

*c* = 0	*X*_1_ → *X*_2_	*X*_2_ → *X*_3_	*X*_3_ → *X*_4_	*X*_4_ → *X*_5_	*X*_2_ → *X*_4_	*X*_5_ → *X*_4_
1A	4.5 / 5.1	5.8 / 5.6	5.6 / 5.4	5.4 / 4.1	4.9 / 4.6	3.8 / 4.8
1B	4.5 / 4.3	5.8 / 5.6	5.9 / 5.5	5.2 / 4.8	4.9 / 4.5	3.9 / 4.4
1C	1.9 / 0.6	2 / 0.5	2.2 / 0.5	2.1 / 0.5	2.2 / 0.4	1.5 / 0.6
2A	4.4 / 4.3	4.8 / 5.5	5.1 / 4.8	4.9 / 4.2	4.7 / 4.4	4.3
2B	3.3 / 2.6	3.6 / 3.3	3.7 / 2.9	3 / 2.9	2.9 / 2.3	3.2 / 3
2C	1 / 0.7	1.4 / 0.3	1.5 / 0.5	1.3 / 0.2	2.1 / 0.6	0.9 / 0.4
2D	1.9 / 0.8	2.4 / 0.9	2.3 / 0.9	1.6 / 0.8	1.7 / 0.6	1.4 / 0.7
*c* = 0.2	**X**_**1**_ → **X**_**2**_	**X**_**2**_ → **X**_**3**_	**X**_**3**_ → **X**_**4**_	**X**_**4**_ → **X**_**5**_	*X*_2_ → *X*_4_	*X*_5_ → *X*_4_
1A	**58** / **100**	**51.8** / **100**	**57** / **100**	**52.7** / **100**	6.5 / 6.6	8.1 / 10.8
1B	**57.5** / **100**	**50.6** / **100**	**54.5** / **100**	**49.2** / **100**	4.9	5.6 / 7
1C	**34.3** / **100**	**17.5** / **100**	**18.9** / **100**	**16.6** / **100**	0.5 / 0	0.5 / 0
2A	**57.1** / **100**	**56.9** / **100**	**62.1** / **100**	**57** / **100**	7.7 / 7.1	8.7 / 11.3
2B	**49.8** / **100**	**52.1** / **100**	**58.1** / **100**	**52.2** / **100**	4.9 / 2.4	6.2 / 4.2
2C	**30.6** / **100**	**24.2** / **99.8**	**26** / **99.9**	**24.3** / **99.8**	0.5 / 0	1 / 0.1
2D	**31.3** / **100**	**34.4** / **100**	**38.9** / **100**	**33.5** / **100**	3.2 / 0.8	3.4 / 0.8
*c* = 0.4	**X**_**1**_ → **X**_**2**_	**X**_**2**_ → **X**_**3**_	**X**_**3**_ → **X**_**4**_	**X**_**4**_ → **X**_**5**_	*X*_2_ → *X*_4_	*X*_5_ → *X*_4_
1A	**100**	**99.7** / **100**	**99.8** / **100**	**99.4** / **100**	14 / 56.8	14.1 / 23
1B	**100**	**99.8** / **100**	**99.6** / **100**	**99.1** / **100**	5.9 / 21.4	5 / 4.7
1C	**100**	**85.2** / **100**	**87.7** / **100**	**84** / **100**	0.4 / 0.6	0.8 / 0.2
2A	**100**	**99.9** / **100**	**100**	**99.8** / **100**	18.2 / 59.5	16.9 / 25
2B	**100**	**99.9** / **100**	**99.9** / **100**	**99.8** / **100**	11 / 26.3	9.6 / 6.4
2C	**99.8** / **100**	**97.1** / **100**	**97.6** / **100**	**95.1** / **100**	1.5 / 2.7	2.4 / 0.3
2D	**99.8** / **100**	**99.1** / **100**	**99.1** / **100**	**98.5** / **100**	5.1 / 9.4	5.1 / 2.2

The results for method 2D are similar to methods 1C and 2C. All the true couplings are well identified, while spurious couplings are found at a percentage higher to 5% only in three instances for *c* = 0.4 and *n* = 2048: *X*_1_ → *X*_3_ (5.8%), *X*_2_ → *X*_4_ (9.4%) and *X*_3_ → *X*_5_ (15.4%).

As resampled time series become less dependent, we observe a loss in the power of the test for *n* = 512, especially when couplings are not very strong. Regarding the size of the test, for *c* = 0.2 the percentage of rejections for indirect (e.g. *X*_2_ → *X*_4_) or no coupling (e.g. *X*_5_ → *X*_4_) is modestly above the 5% level only for 1A and 2A, while for *c* = 0.4 is substantially higher for 1A and 2A and lower for 1B and 2B. For example, we obtain for scheme 1A and *n* = 2048: 50.5% for *X*_1_ → *X*_3_ (indirect coupling), 22.2% for *X*_2_ → *X*_1_ (no coupling), 56.8% for *X*_2_ → *X*_4_ (indirect coupling), 19.7% for *X*_3_ → *X*_2_ (no coupling), 62.2% for *X*_3_ → *X*_5_ (indirect coupling), 22.9% for *X*_4_ → *X*_3_ (no coupling) and 14.1% for *X*_5_ → *X*_4_ (no coupling). Respective results are indicated by the scheme 2A. When considering more independent resampled time series, the corresponding percentages of indirect and no couplings decrease, e.g. for method 1B and *n* = 2048: 27.5% for *X*_1_ → *X*_3_, 20% for *X*_2_ → *X*_1_, 21.4% for *X*_2_ → *X*_4_, 3.7% for *X*_3_ → *X*_2_, 28% for *X*_3_ → *X*_5_, 4.1% for *X*_4_ → *X*_3_ and 4.7% for *X*_5_ → *X*_4_. Similar results are observed for 2A. The correct test size, i.e. the probability of falsely rejecting the null hypothesis being close to *α* = 0.05, is attained only with the resampling methods of type C; the percentage of the significant PTE values for the uncoupled cases varies from 0% to 4.7% for both 1C and 2C and both *n*, while spurious causality is detected for cases A and B. As *n* and *c* increase, the percentage of those spurious indications increases.

## Application

**Fig 4:**
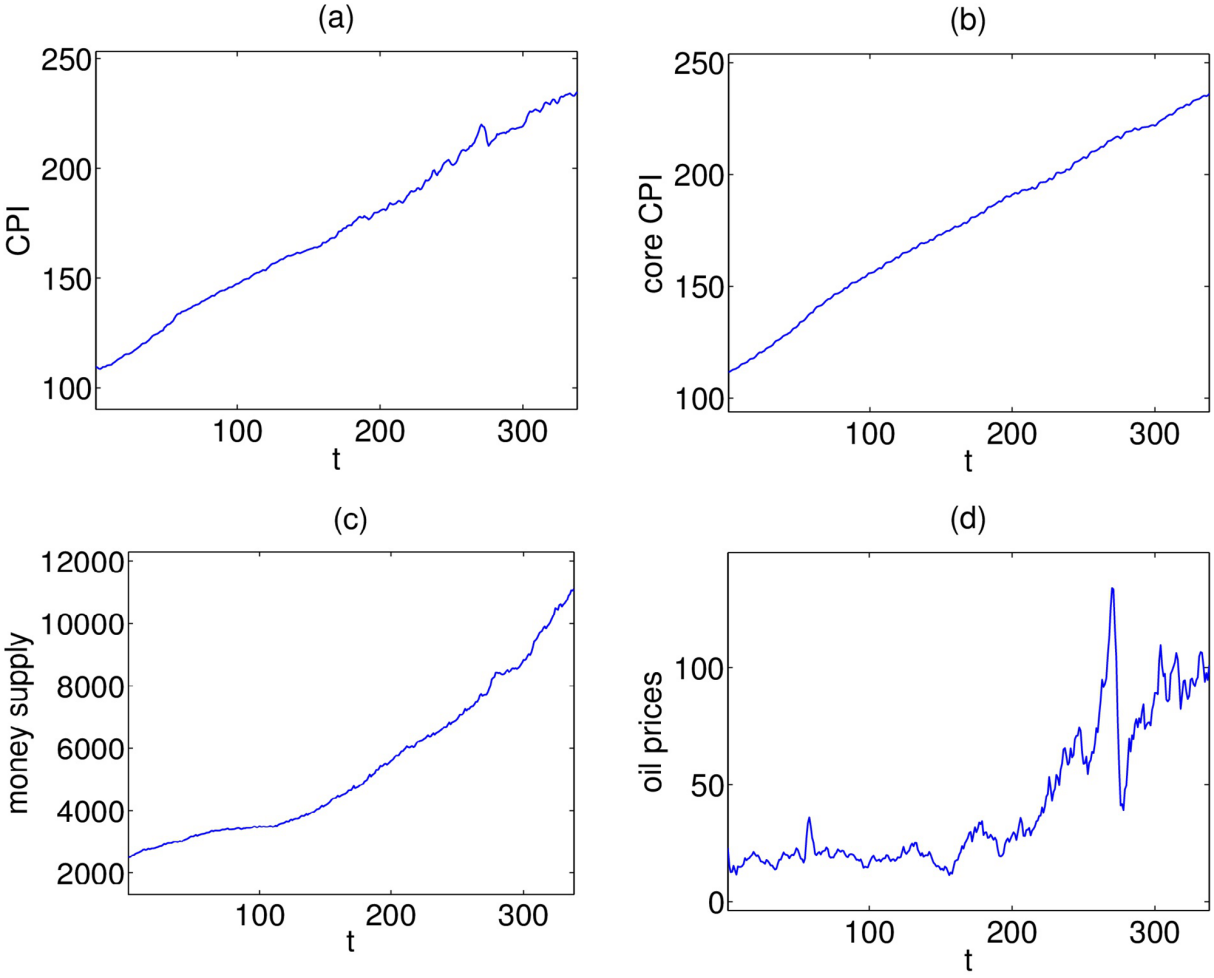
Monthly observations of the (a) CPI, (b) core CPI, (c) money supply and (d) oil prices, respectively.

In the effort to provide further evidence on the possible sources of US inflation in the post-Volcker era, we will try to gain insights from the application of the PTE by employing the aforementioned resampling methods. For this reason, we create two 3-variate systems of real economic variables, the first one consisting of monthly observations for the US Consumer Price Index for All Urban Consumers (CPI), the money supply (M2, Billions of Dollars) and the crude oil prices (West Texas Intermediate—Cushing, Oklahoma, Dollars per Barrel) while the second one is obtained by replacing CPI with the core CPI (Fig 4). The data are not seasonally adjusted and the sample spans from 01-01-1986 to 01-02-2014. We used the longer available sample at the time the application is implemented in order to ensure PTE accuracy. Since in the post-2009 period, US inflation reached very low values in association with the QE strategy of the Federal Reserve, we strongly believe that our findings over the period of interest (i.e. before the crisis of 2007–2009) are not qualitatively affected. To assess the impact of restricting the sample until 2009, we re-estimated the PTE for both systems. In the first case, we observe a feedback between CPI inflation and crude oil changes, while for the 2nd system identical causal relationships appear. Prices are transformed into growth rates by using their first logarithmic differences to give inflation (*Y*_1_) in the case of CPI, core inflation (*Y*_11_), M2 returns (*Y*_2_) and oil price changes (*Y*_3_).

For the assessment of the statistical significance of the PTE we look back at the seven resampling methods mentioned above. The embedding dimension *m* for the estimation of the PTE is set equal to one (*m* = 1), often used in log differenced data expecting to have very short memory [59] and the number of nearest neighbors is ten (*k* = 10).

The empirical findings from the application of the PTE on the 1st 3-variate system consistently reveal the coupling oil (*Y*_3_) → inflation (*Y*_1_). The fact that this linkage becomes statistically insignificant when the core CPI inflation is taken into account, is an indication that the observed inflation in the post-1986 period cannot be interpreted with means of traditional cost-push mechanisms. Table 8 displays the connectivity results based on each of the seven resampling methods, where statistically significant probabilities are given in bold (when *p*-value <0.05). Aside from the link *Y*_3_ → *Y*_1_, few additional links also appear. Since we do not obtain any consistency, these sparse links may be due to estimation biases, the method of assessing its statistical significance or existence of high noise in the data. Table 9 presents the results for the 2nd 3-variate system, where the CPI inflation has been replaced by the core CPI inflation. As it can been seen, the influence of crude oil to core inflation is not statistically significant and new relationships emerge. We detect a persistent causal feedback between core inflation (*Y*_11_) and money supply (*Y*_2_), while oil (*Y*_3_) causes money supply (*Y*_2_). The former bidirectional causality underscores the role of US monetary policy in controlling inflation during Great Moderation. This has been achieved through changes in the policy rate and monetary authority open market operations. Thus, via buying and selling bonds in the market, the Federal Reserve adjusted monetary base that in turns affected accordingly the monetary aggregates, such as M2. The latter unidirectional link from oil to money supply is in line with [60] conclusion that the demand-fuelled oil price rises in the 2000s have been accommodated by economic policy.

**Table 8:** The *p*-values from PTE based on the seven resampling methods for the 1st 3-variate system including the CPI inflation. Conditioning on the third variable is implied. Significant causal effects are denoted in bold.

*p*-value	*Y*_1_ → *Y*_2_	*Y*_2_ → *Y*_1_	*Y*_2_ → *X*_3_	*Y*_3_ → *Y*_2_	*Y*_1_ → *X*_3_	*Y*_3_ → *Y*_1_
1A	0.4211	0.3323	0.2533	0.0560	**0.0363**	**0.0067**
1B	0.0757	**0.0165**	0.3816	**0.0165**	0.9933	**0.0165**
1C	0.5296	0.2928	0.2435	0.2139	**0.0363**	**0.0067**
2A	0.1744	0.1152	0.2040	0.2435	0.0856	**0.0067**
2B	0.2928	0.2237	0.2336	0.3323	0.1053	**0.0165**
2C	0.1645	0.1053	0.3224	0.3224	0.0659	**0.0067**
2D	0.2237	0.1349	0.1843	0.2435	0.0757	**0.0461**

**Table 9:** As Table 8 but for the 2nd 3-variate system including the core CPI inflation.

*p*-value	*Y*_11_ → *Y*_2_	*Y*_2_ → *Y*_11_	*Y*_2_ → *X*_3_	*Y*_3_ → *Y*_2_	*Y*_11_ → *X*_3_	*Y*_3_ → *Y*_11_
1A	**0.0363**	**0.0067**	0.3816	**0.0461**	0.4507	0.3816
1B	0.9933	**0.0067**	**0.0067**	**0.0067**	**0.0067**	**0.0067**
1C	0.2040	0.0659	0.4803	**0.0264**	0.5888	0.3915
2A	**0.0165**	**0.0067**	0.4013	**0.0067**	0.6776	0.3816
2B	**0.0067**	**0.0067**	0.6480	**0.0461**	0.7171	0.6184
2C	**0.0067**	**0.0067**	0.5592	**0.0165**	0.6381	0.5000
2D	**0.0363**	**0.0067**	0.3816	0.3816	0.4507	0.3816

The relationship between crude oil and consumer price index has been determined dynamically over the past 50 years. The strength of the linkage seems to vary conditionally to several factors including the nature of oil shocks, the response of monetary policy and the rigidities in the labor market. In the 1970s the oil price shocks of 1973 and 1979 were associated with significant reductions in OPEC supply. In the early of middle 1980s starts a phase of stability for the US economy, known as The Great Moderation, characterized by low volatility in inflation and output. Oil prices however become more volatile again from the second half of the 1990s until mid-2008. While the oil shock episodes in 1973 and 1979 coincide with an increase in the US inflation and the beginning of rising unemployment, the variation of these two variables becomes smaller in size during the episodes of 1999–2000 and 2002–2007.

Whereas the stable core CPI in the post-1984 period, [61] show that the relative contribution of oil shocks to CPI inflation has increased since oil price changes have passed through the energy component of CPI. This lack of significant second-round effects on core inflation via cost-push mechanisms puts forward the difference in the effects of oil prices in the 1970s and the 2000s. Oil prices are not only affected by disturbances in supply. Oil shocks can be the consequence of technological changes or financial innovation able to affect consumers’ demand for oil. According to [62] the oil price increase between 2009 and mid-2008 was driven by global demand shocks and as such it was not associated with recessionary dynamics of the US economy. Going further, [63] defines oil price fluctuations as symptoms of the underlying oil demand and oil supply shocks and conclude that disentangling between these two sources can prevent from unnecessary monetary policy interventions.

## Conclusion

This study stems from the necessity to derive an effective causality test for the investigation of the connectivity structure of a multivariate complex system. Specifically, we investigate how the performance of a (direct) causality test is affected by the scheme generating the resampled data [29, 39, 47]. Our contribution is two-fold, with respect to the methodology and the application. Regarding the methodology, we introduce new resampling methods for the non-causality test. Regarding the application, we obtain coherent results based on the partial transfer entropy (PTE) and all the aforementioned resampling methods, highlighting the complex nature of oil shocks through their impact on inflation.

The importance of assessing the statistical significance for the partial transfer entropy (PTE) has been explored via a simulation study. In the absence of direct coupling *X* → *Y*|*Z*, by definition, the mutual information of *X* and *Y* conditioned on *Z* should be theoretically zero, i.e. *I*(*Y*;*X*|*Z*) = 0. The formulation of more independent resampled data (settings B and C) compared to the standard technique (setting A), all consistent to the null hypothesis *I*(*Y*;*X*|*Z*) = 0, seems to account better for the bias of the test statistic and helps prevent false detection of coupling in the case of the nonlinear coupled systems. The size and the power of the test are improved with settings B and C, especially if the direct couplings are strong. However, for large *n* and *c*, settings B and C may also give spurious couplings, such as for *X*_2_ → *X*_4_ for System 3. We should also underline that the performance of PTE is affected by the number of observed variables [53]. On the other hand, when the coupled system is linear, independence setting A seems to be more efficient in identifying weak couplings. The method 2D is also effective for the nonlinear simulation systems and less effective for the linear coupled system, detecting spurious couplings.

It turns out that the PTE estimated on resampled time series increases with increasing level of randomness; i.e. the surrogate PTE values increase going from setting A to C. In addition, the spread of the surrogate PTE distribution gets larger, implying that smaller PTE values on the original time series are likely to be found statistically not significant and consequently less spurious couplings are detected. Figs 1–3 display the distribution of the surrogate PTE values for systems 1 and 2 with respect to each resampling scheme in order to visualize these findings. When we detect the true causality with high probability, we may also get spurious couplings. In order to avoid the detection of false connectivity, we may have a loss in sensitivity. This higher specificity comes at the cost of lower sensitivity, and vice versa. Thus, optimality is not achieved for any of the first six resampling methods, but it becomes clear that the significance test for the PTE gets more conservative as resampling is more random. Regarding method 2D, the bootstrap PTE values are centered by construction around zero and therefore it focuses on the spread of the distribution of the PTE on the bootstrapped data rather than the bias. For linear systems, the bias is larger and method 2D performs worse.

We note that the seven resampling methods have comparable computational cost as randomization procedures are involved at all cases in the same way. Further, they can be utilized for any test statistic in order to examine the null hypothesis of no causal effects. Ongoing research aims at further investigating the performance of various causality measures, gaining insight from the significant impact that the selection of alternative resampling techniques may have.

In the context of the application, using the PTE with all examined statistical significance tests, we confirm the stability of core inflation over the post-Volcker era including the period of Great Moderation. The strong causal influence of crude oil on the total CPI inflation and the absence of link with the core CPI inflation clearly highlight the contribution of oil demand shocks as opposite to the oil supply shocks in the 2000s that the US economy experienced in the 1970s.

## Supporting information

S1 Table Percentage of significant PTE values for system 3 for *n* = 512/2048, for all resampling methods. A single number is displayed when the same percentage corresponds to both *n*. The true couplings are highlighted.
(DOCX)

S1 Dataset The matlab codes for generating the corresponding simulation time series of the manuscript are provided as a Supplementary File. The financial time series from the real applications can be downloaded from the Federal Reserve Bank of Saint Louis at the following link: https://fred.stlouisfed.org/categories.
(ZIP)
