# Supplementary material for: Assessment of resampling methods for causality testing: A note on the US inflation behavior
Source: PLoS One. 2017 Jul 14;12(7):e0180852. doi: 10.1371/journal.pone.0180852 (PMC5510825; doi:10.1371/journal.pone.0180852)
Supplement: S1 Table — A single number is displayed when the same percentage corresponds to both n. The true couplings are highlighted. (DOCX) [file pone.0180852.s001.docx]

**S1 Table.** Percentage of significant PTE values for system 3 for n = 512 / 2048, for all resampling methods. A single number is displayed when the same percentage corresponds to both n. The true couplings are highlighted.

| **c=0** | **X1→X2** | **X2→X3** | **X3→X4** | **X4→X5** | **X1→X3** | **X1→X4** | **X1→X5** | **X2→X1** | **X2→X4** | **X2→X5** |
| --- | --- | --- | --- | --- | --- | --- | --- | --- | --- | --- |
| **1A** | 4.5/5.1 | 5.8/5.6 | 5.6/5.4 | 5.4/4.1 | 4.3/5.8 | 4.6/5.4 | 5.2/4.5 | 5.8/4.3 | 4.9/4.6 | 5.2/4.7 |
| **1B** | 4.5/4.3 | 5.8/5.6 | 5.9/5.5 | 5.2/4.8 | 4.1/5.7 | 4.8/4.2 | 5.3/4.1 | 5.1/4.7 | 4.9/4.5 | 4.8/4.8 |
| **1C** | 1.9/0.6 | 2/0.5 | 2.2/0.5 | 2.1/0.5 | 1.3/0.6 | 1.6/0.8 | 2/0.8 | 2.3/0.3 | 2.2/0.4 | 2.2/0.4 |
| **2A** | 4.4/4.3 | 4.8/5.5 | 5.1/4.8 | 4.9/4.2 | 4.4/5.1 | 4.9/4.6 | 5/3.9 | 5.1/3.9 | 4.7/4.4 | 3.9/4.5 |
| **2B** | 3.3/2.6 | 3.6/3.3 | 3.7/2.9 | 3/2.9 | 2.5/2.8 | 3.9/2.6 | 3.6/2.2 | 3/2.5 | 2.9/2.3 | 3.1/2.8 |
| **2C** | 1/0.7 | 1.4/0.3 | 1.5/0.5 | 1.3/0.2 | 1.2/0.5 | 2/0.4 | 1.7/0.5 | 1.8/0.6 | 2.1/0.6 | 1.3/0.6 |
| **2D** | 1.9/0.8 | 2.4/0.9 | 2.3/0.9 | 1.6/0.8 | 1.5/1 | 1.7/0.8 | 1.7/0.9 | 1.8/0.6 | 1.7/0.6 | 1.5/0.9 |
|  | **X3→X1** | **X3→X2** | **X3→X5** | **X4→X1** | **X4→X2** | **X4→X3** | **X5→X1** | **X5→X2** | **X5→X3** | **X5→X4** |
| **1A** | 5/3.9 | 4.6/5.2 | 4.3/5.0 | 4.7/5.8 | 4.5/4.8 | 4.2/5.7 | 3.5/4.8 | 5.2/5.1 | 4.4/4.7 | 3.8/4.8 |
| **1B** | 5.3/4.8 | 4.9/5.2 | 4.4/4.9 | 4.4/5.6 | 4.6/4.2 | 3.9/6 | 4.4/5.2 | 5/5 | 4.7/5.5 | 3.9/4.4 |
| **1C** | 2.1/0.4 | 1.8/0.6 | 1.2/0.5 | 2.4/0.7 | 1.6/0 | 1.8/0.8 | 2.2/0.3 | 1.9/0.3 | 1.5/0.7 | 1.5/0.6 |
| **2A** | 4.5/3.5 | 4.8/5.2 | 4/4.3 | 4.5/4.9 | 4.1/4.3 | 3.1/6.2 | 3.7/4.7 | 4.6/4.3 | 3.9/4.4 | 4.3/4.3 |
| **2B** | 3.3/2.6 | 3.2/2.8 | 2.8/2.7 | 3.4/2.8 | 2.4/2.5 | 2.8/3.1 | 2.3/2.9 | 3.7/2.2 | 2.7/3.1 | 3.2/3 |
| **2C** | 1.1/0.4 | 1.2/0.6 | 0.9/0.5 | 1.6/0.8 | 1.3/0.1 | 1.9/0.8 | 0.9/0.1 | 1.3/0.1 | 1.4/0.8 | 0.9/0.4 |
| **2D** | 2/0.9 | 1.6/0.9 | 1.4/0.7 | 2.1/1 | 1.4/0.4 | 1.6/1.4 | 1.2/0.2 | 1.2/0.5 | 1.3/1.3 | 1.4/0.7 |
| **c=0.2** | **X1→X2** | **X2→X3** | **X3→X4** | **X4→X5** | **X1→X3** | **X1→X4** | **X1→X5** | **X2→X1** | **X2→X4** | **X2→X5** |
| **1A** | **58/100** | **51.8/100** | **57/100** | **52.7/100** | 5.3/6.4 | 5.2/5.8 | 5.5/5.8 | 6.3/13.6 | 6.5/6.6 | 5.8/5.2 |
| **1B** | **57.5/100** | **50.6/100** | **54.5/100** | **49.2/100** | 5.3/4.2 | 4.5/3.9 | 4.3/4 | 5.6/14.4 | 4.9 | 4.7/3.6 |
| **1C** | **34.3/100** | **17.5/100** | **18.9/100** | **16.6/100** | 1.2/0.1 | 1.5/0.2 | 0.6/0.1 | 2.7/1.8 | 0.5/0 | 0.5/0 |
| **2A** | **57.1/100** | **56.9/100** | **62.1/100** | **57/100** | 5.3/5.7 | 4.4/5.3 | 4.7/4.5 | 5/13 | 7.7/7.1 | 6.2/5.3 |
| **2B** | **49.8/100** | **52.1/100** | **58.1/100** | **52.2/100** | 2.1/1.7 | 2.4/1.2 | 2.2/1.6 | 3.1/7.8 | 4.9/2.4 | 4.5/1.4 |
| **2C** | **30.6/100** | **24.2/99.8** | **26/99.9** | **24.3/99.8** | 0.7/0 | 0.5/0 | 0.3/0.1 | 1.4/2.1 | 0.5/0 | 0.7/0 |
| **2D** | **31.1/100** | **34.4/100** | **38.9/100** | **33.5/100** | 1/0 | 1.6/0.2 | 1.3/0.3 | 2.1/2.7 | 3.2/0.8 | 2/0.2 |
|  | **X3→X1** | **X3→X2** | **X3→X5** | **X4→X1** | **X4→X2** | **X4→X3** | **X5→X1** | **X5→X2** | **X5→X3** | **X5→X4** |
| **1A** | 5.7/3.9 | 8/9 | 6.7/8.7 | 5.6/5 | 4.9/4.9 | 8.5/9.6 | 6.1/4.1 | 6.2/4.3 | 4.8/5.1 | 8.1/10.8 |
| **1B** | 4.8/3.8 | 5.8/5.4 | 5.3/4.8 | 4.7/3.9 | 3.1/3 | 6.2/6.3 | 5.4/4.1 | 3.8/3.1 | 3.5/2.8 | 5.6/7 |
| **1C** | 1.8/0.2 | 0.8/0 | 0.2/0 | 1.6/0.1 | 0.3/0 | 1/0 | 2.3/0.1 | 0.1/0 | 0.5/0 | 0.5/0 |
| **2A** | 3.9/3.3 | 9/8.3 | 7/7.6 | 5.2/4 | 5.9/5.3 | 9.9/11.1 | 4.1/3.8 | 6.4/4.6 | 5.4/5.5 | 8.7/11.3 |
| **2B** | 2.7/1.2 | 5.5/2.2 | 5.4/3.1 | 3.7/1.7 | 3.8/1.7 | 7.3/3.6 | 3.2/1.5 | 3.6/0.8 | 4.5/1.5 | 6.2/4.2 |
| **2C** | 0.9/0.3 | 0.6/0.1 | 0.4/0 | 1.2/0 | 0.2/0 | 0.9/0 | 1.5/0 | 0.6/0 | 0.3/0 | 1/0.1 |
| **2D** | 1.8/0.3 | 2.6/0.5 | 2.5/0.8 | 1.9/0 | 1.4/0.4 | 2.4/0.6 | 2.4/0.2 | 1.8/0.2 | 1.8/0 | 3.4/0.8 |
| **c=0.4** | **X1→X2** | **X2→X3** | **X3→X4** | **X4→X5** | **X1→X3** | **X1→X4** | **X1→X5** | **X2→X1** | **X2→X4** | **X2→X5** |
| **1A** | **100** | **99.7/100** | **99.8/100** | **99.4/100** | 10.2/50.5 | 6.6/5.4 | 5.5/4.2 | 4.2/22.2 | 14/56.8 | 5.6/8.7 |
| **1B** | **100** | **99.8/100** | **99.6/100** | **99.1/100** | 6/27.5 | 3.5/1.5 | 2.9/0.8 | 2.9/20 | 5.9/21.4 | 1.3/0.8 |
| **1C** | **100** | **85.2/100** | **87.7/100** | **84/100** | 1.7/4.7 | 0.9/0 | 0.4/0 | 1.2/2.8 | 0.4/0.6 | 0/0 |
| **2A** | **100** | **99.9/100** | **100** | **99.8/100** | 8.1/47.9 | 5.2/4.9 | 4.4/4.1 | 2.8/20.5 | 18.2/59.5 | 8.1/9.2 |
| **2B** | **100** | **99.9/100** | **99.9/100** | **99.8/100** | 3.9/19 | 1.7/0.5 | 1.2/0.1 | 1.8/12.9 | 11/26.3 | 3.6/1.2 |
| **2C** | **99.8/100** | **97.1/100** | **97.6/100** | **95.1/100** | 1.1/3.4 | 0.4/0 | 0.3/0 | 0.5/2.3 | 1.5/2.7 | 0.4/0 |
| **2D** | **99.8/100** | **99.1/100** | **99.1/100** | **98.5/100** | 2/5.8 | 1/0 | 0.7/0 | 1/3.4 | 5.1/9.4 | 1.2/0.5 |
|  | **X3→X1** | **X3→X2** | **X3→X5** | **X4→X1** | **X4→X2** | **X4→X3** | **X5→X1** | **X5→X2** | **X5→X3** | **X5→X4** |
| **1A** | 3.8/4.8 | 10.1/19.7 | 14/62.2 | 4.8/3.7 | 3.3/5.7 | 12.4/22.9 | 4.2/4.5 | 4.1/6 | 4.7/9 | 14.1/23 |
| **1B** | 3.1/3.7 | 4.2/3.7 | 6.4/28 | 3.7/3.2 | 1.1/0.8 | 3.5/4.1 | 3.3/4 | 1.5/0.9 | 1.5/0.9 | 5/4.7 |
| **1C** | 0.4/0.2 | 0.3/0 | 0.4/0**.**6 | 1/0.2 | 0/0 | 0.9/0 | 0.9/0.5 | 0/0 | 0.3/0 | 0.8/0.2 |
| **2A** | 2.5/3.6 | 12.2/18 | 18.8/65.3 | 3.5/2.8 | 4.5/5.7 | 16/25.6 | 3.3/4 | 4.5/5.5 | 8.8/10.2 | 16.9/25 |
| **2B** | 1.6/1.8 | 5.6/3 | 12.1/32.6 | 1.5/1.6 | 0.9/0.5 | 7.5/5.7 | 1.7/1.8 | 2/0.3 | 4.2/0.9 | 9.9/6.4 |
| **2C** | 0.4/0.2 | 0.4/0 | 1.3/2.6 | 0.6/0 | 0.1/0 | 1.5/0.2 | 0.5/0.2 | 0.1/0 | 0.5/0 | 2.4/0.3 |
| **2D** | 1/0.6 | 2.6/0.9 | 7/15.4 | 1/0.3 | 0.4/0.3 | 3.4/1.2 | 1/0.7 | 0.9/0 | 1.4/0.3 | 5.1/2.2 |
